# An open-source UFBGA *µ*-board for wearable devices

**DOI:** 10.1016/j.ohx.2022.e00281

**Published:** 2022-02-19

**Authors:** Rui Azevedo Antunes, Luís Brito Palma

**Affiliations:** a ESTSetúbal and CIIAS, Polytechnic Institute of Setúbal, Centre of Technology and Systems – Uninova, Monte da Caparica, Portugal; b Department of Electrical and Computer Engineering, NOVA School of Science and Technology, Centre of Technology and Systems – Uninova, Monte da Caparica, Portugal

**Keywords:** UFBGA, PCB hardware manufacturing, AVR microcontroller, Instrumentation, Health tech

## Abstract

This paper presents the development of an open-source, low-sized, BGA microcontroller breakout board, that can be used for the development of wearable and cyber-physical prototypes. The board is based on the low power, 8-bit, ATtiny20-CCU Microchip AVR microcontroller. The ATtiny20-CCU can be programmed without bootloader, using the Atmel Tiny Programming Interface (TPI), instead of In-System Programming (ISP). The C code used to program the microcontroller can be written and compiled using the Microchip Studio freeware platform. The ATtiny20-CCU Ultra Fine-pitch Ball Grid Array (UFBGA) packaging technology allows the shrinkage of the conceived Electroless Nickel-Immersion Gold (ENIG) Printed Circuit Board (PCB) to a size of only 15*.*5 × 13 mm. Its low cost also makes it a viable option for developing many educational electronic projects, especially for Instrumentation and Assistive Technology. The contribution of this paper is mainly the hardware prototype design, the PCB manufacturing, building and test of a very low-sized open source *µ*-breakout PCB board, for wearable Instrumentation applications, towards the emergent Society/Industry 5.0.

## Graphical abstract

**Figure:**
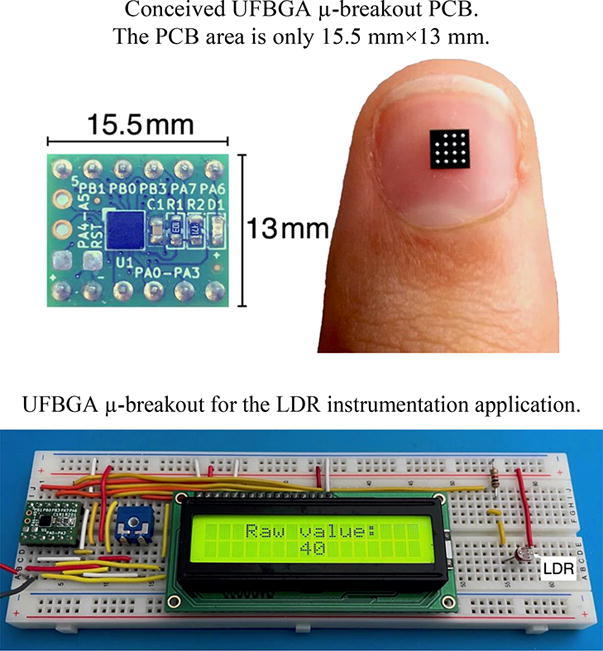

**Specifications table**

**Table:** 

**Hardware Name**	UFBGA *µ*-board for wearable devices
**Subject Area**	• General (PCB Hardware Manufacturing)
	• Instrumentation
	• Alternatives to Existing Instrumentation Infrastructure
	• Educational Tools and Open Source
	• Engineering and Material Science
	• Medical
**Hardware type**	• Field measurements and sensors
	• Measuring physical properties and in-lab sensors
	• Electrical engineering and computer science
**Closest Commercial Analog**	No commercial analog is available
**Open Source License**	Creative Commons Attribution-ShareAlike license
**Cost of Hardware**	*≈* 5 € (Euro)
**Source File Repository Source File Repository**	https://doi.org/10.17605/OSF.IO/YG653

## Hardware in context

**Fig. 1:**
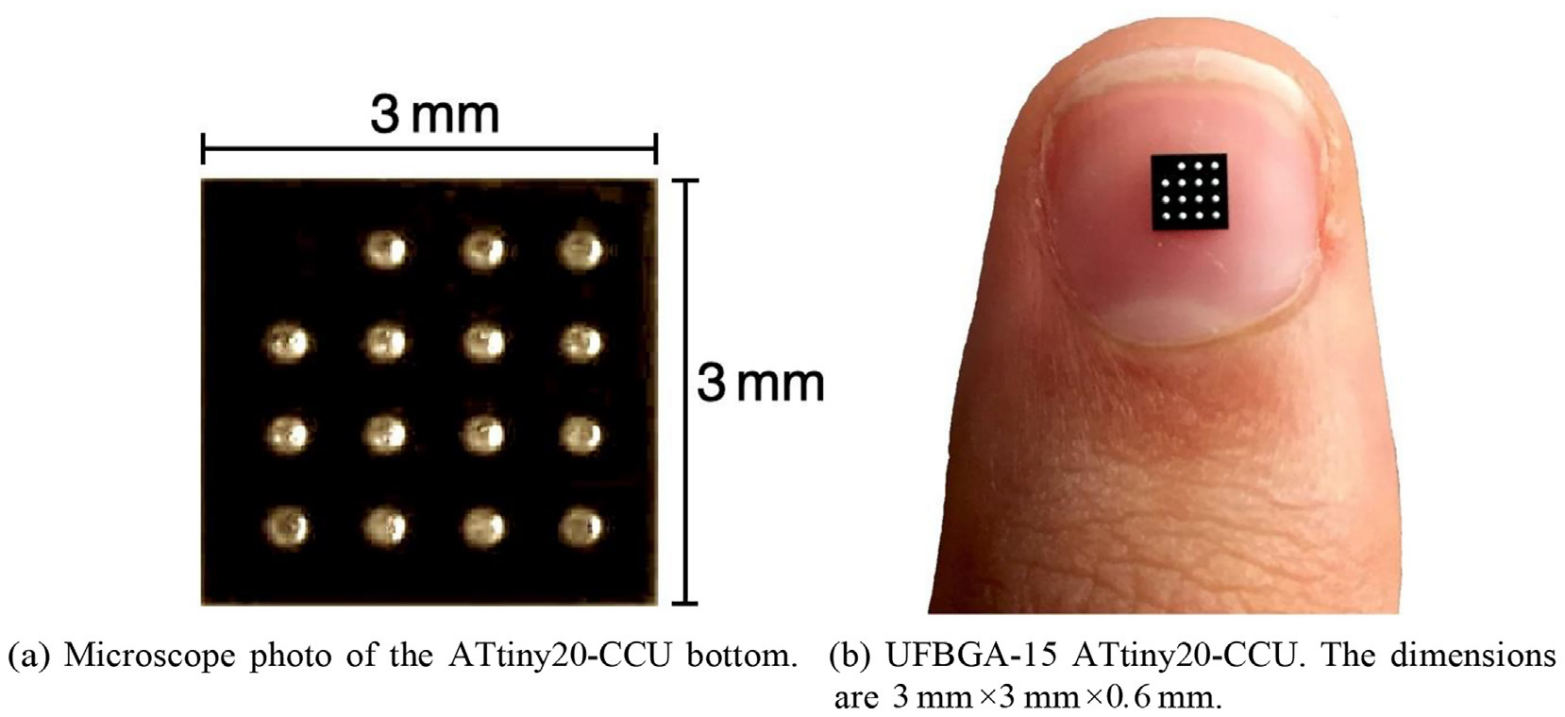
ATtiny20-CCU IC microcontroller.

Nowadays there is an increasing demand for developing light weight, thin, high density and high-performance wearable hardware/software for Instrumentation and/or non-invasive healthcare applications. Recent advances in the electronics fabrication industry, due to the introduction of BGA (Ball Grid Array) chip high-density packaging technology, led the ratio between chip area and package area to be very close to 1. This is mandatory for the design of new assistive devices towards the resilient, human-centred and inclusive Society 5.0 [1], [2], [3], “a human-centred society that balances economic advancement with the resolution of social problems by a system that highly integrates cyberspace and physical space” [4]. BGA is a surface mount technology, in which there are employed ball-shape bumps at the bottom of a package IC (Integrated Circuit) (Fig. 1), instead of perimeter contact pads, aligned and soldered to the Printed Circuit Board.

Fine-pitch BGA packaging technology (FBGA), used nowadays for tiny wearable device fabrication surpasses the popular QFN (Quad Flat No-lead) packaging technology, due to the following advantages:

• Higher number of Inputs/Outputs can be supported;
• Larger spaces between soldering joints makes easy the automatic amplified alignment;
• Higher packaging and reduced soldering imperfections;
• Coplanarity is achieved, because after the solder being melted, it will automatically correct any planarity drifts;
• High reliability, due to the automatic self-alignment tension effect in the soldering joints;
• Better electrical and frequency behaviour, due to smaller soldering joints and lower inductance.

Ultra Fine-pitch Ball Grid Array (UFBGA) packaging technology is essentially a Fine-pitch BGA (FBGA) variant, with maximum package thickness of 0*.*65 mm. Typical body Sizes for FBGA are between 3*×*3 mm and 23*×*23 mm. The FBGA ball pitch varies between 0*.*4 and 1*.*0 mm (the ATtiny20-CCU UFBGA has a ball pitch of 0*.*65 mm). Present FBGA variants are: LFBGA (Low-Profile Fine-pitch Ball Grid Array), TFBGA (Thin Profile Fine-pitch Ball Grid Array), VFBGA (Very Thin Profile Fine-pitch Ball Grid Array), WFBGA (Very-Very Fine-pitch Ball Grid Array), Ultra Fine-pitch Ball Grid Array (UFBGA) and XFBGA (Extremely Thin Fine-pitch Ball Grid Array). Nowadays, typical maximum IC FBGA package thickness are 1*.*70 mm (LFBGA), 1*.*2 mm (TFBGA), 1*.*0 mm (VFBGA), 0*.*8 mm (WFBGA), 0*.*65 mm (UFBGA) and 0*.*50 mm (XFBGA). The ATtiny20-CCU maximum thickness is 0*.*6 mm.

In large FBGA ICs the total number of FBGA balls can reach 450 [5]. The repairing process of replacing all the soldered balls on the BGA chip is designated by *Reballing*. A flip-chip BGA [6] is a particular Ball Grid Array that employs a controlled collapse chip connection. The radiofrequency characteristics of BGA transition with a frequency range from 100 MHz to 40 GHz are analysed in [7]. Recent fabrication strategies for new biosensors on chip solutions can be found in [8]. A relevant low temperature solder process BGA development for large size BGA packages is presented in [9]. Varied Ball BGA technology [10] is a state-of-the-art BGA improvement, to eliminate solder ball bridging defects in Surface-Mount Technology (SMT). Wafer-Level Chip Scale Packaging (WLCSP) [11] is presently the smallest available package in the IC Industry, which can be handled in the same way as BGA.

## Hardware description

The hardware for this tiny, low-cost, developed breakout microcontroller board, consists of a 2-layer Electroless Nickel-Immersion Gold (ENIG) Printed Circuit Board (PCB), with 2.54 mm pin male header pins, to allow breadboard mounting electronic experiments. At the core of this PCB is the Microchip ATtiny20-CCU low-power 8-bit microcontroller, with Ultra Fine-pitch Ball Grid Array (UFBGA) packaging technology.

The ATtiny20-CCU microcontroller [12] has the following features:

• Low cost;
• 2 K bytes of in-system programmable flash, and 128 bytes of SRAM;
• 12 general purpose I/O (Input/Output) lines;
• 8-bit and 16-bit timers/counters, both with two PWM channels;
• 16 general purpose working registers;
• An eight-channel, 10-bit Analogue-to-Digital Converter (ADC);
• A programmable watchdog timer with internal oscillator;
• A slave two-wire interface, and a master/slave Serial Peripheral Interface (SPI);
• Internal and external interrupts;
• Four software selectable power saving modes, and security features;
• An internal calibrated oscillator.

The ATtiny20-CCU operating voltage VCC is from 1*.*8 V to 5*.*5 V and the programming voltage is 5 V. Three speed grade modes are available: 0–4 MHz (1*.*8–5*.*5 V); 0–8 MHz (2*.*7–5*.*5 V) and 0–12 MHz (4*.*5–5*.*5 V). Concerning the power consumption, there are three modes available: the active mode (200 µA, at 1 MHz and 1*.*8 V), the idle mode (25 µA, at 1 MHz and 1*.*8 V) and the power-down mode (*<*0.1 µA, at 1*.*8 V).

**Fig. 2:**
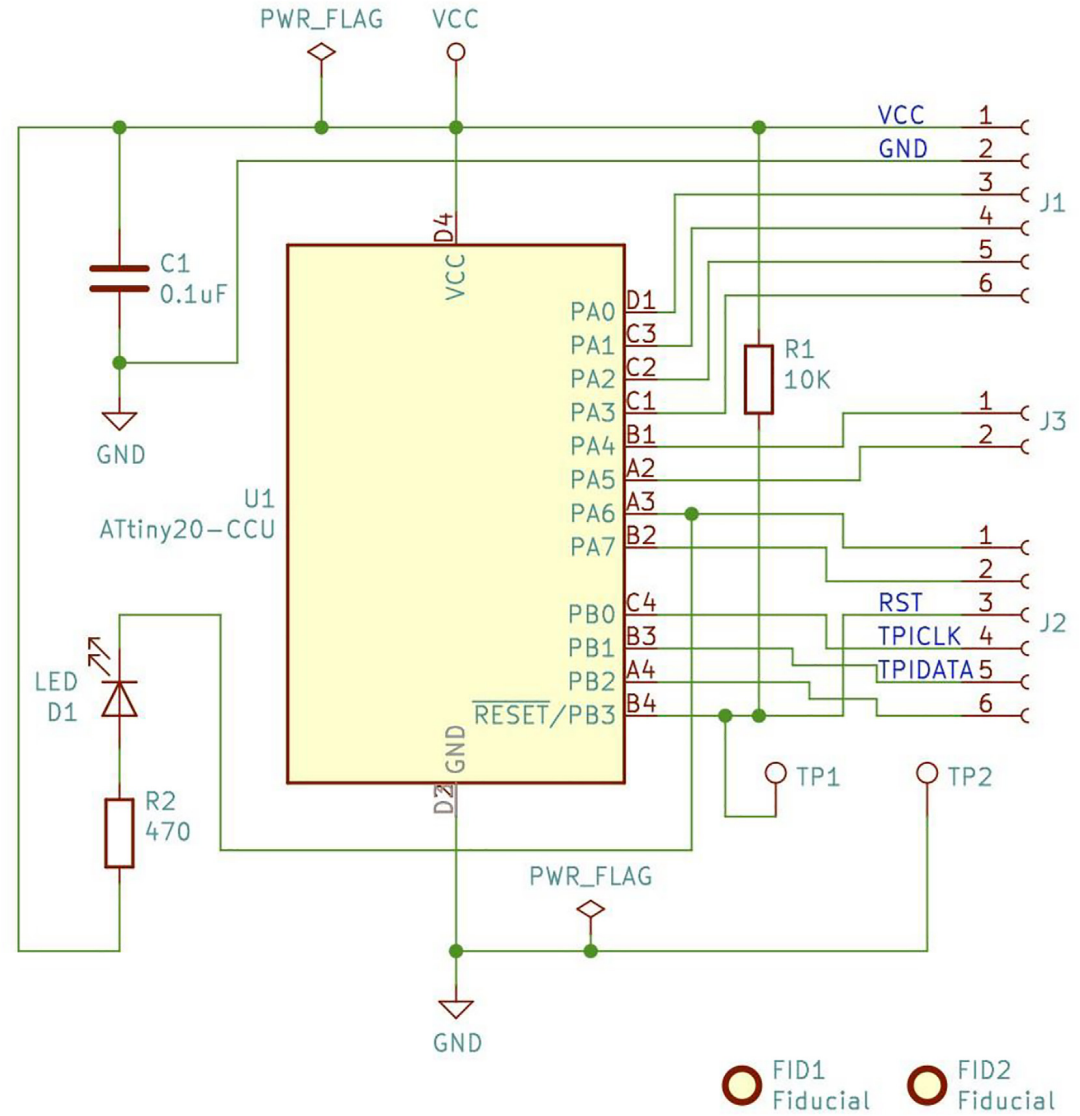
The Ultra Fine-pitch Ball Grid Array (UFBGA) *µ*-breakout board KiCad schematics.

In Fig. 2 the schematics circuit for the UFBGA *µ*-board is shown. To reduce the cost, the number of electronic components was designed to be minimal (only four): a 0.1 µF capacitor (C1) to suppress the power noise in the microcontroller; a pull-up 10 kΩ resistor (R1) to allow a microcontroller reset, by short-circuiting the test-point PCB pads, and the D1 red LED, in serial with the 470 Ω resistor (R2), to allow a built-in functional LED test.

The following list summarizes the main benefits of the UFBGA *µ*-breakout developed board:

• Low-cost and low-power board for many instrumentation applications;
• Small size and light weight, suitable for wearable health tech devices;
• Open-source PCB design, easy to build with Printed Circuit Board Assembly (PCBA).

**Fig. 3:**
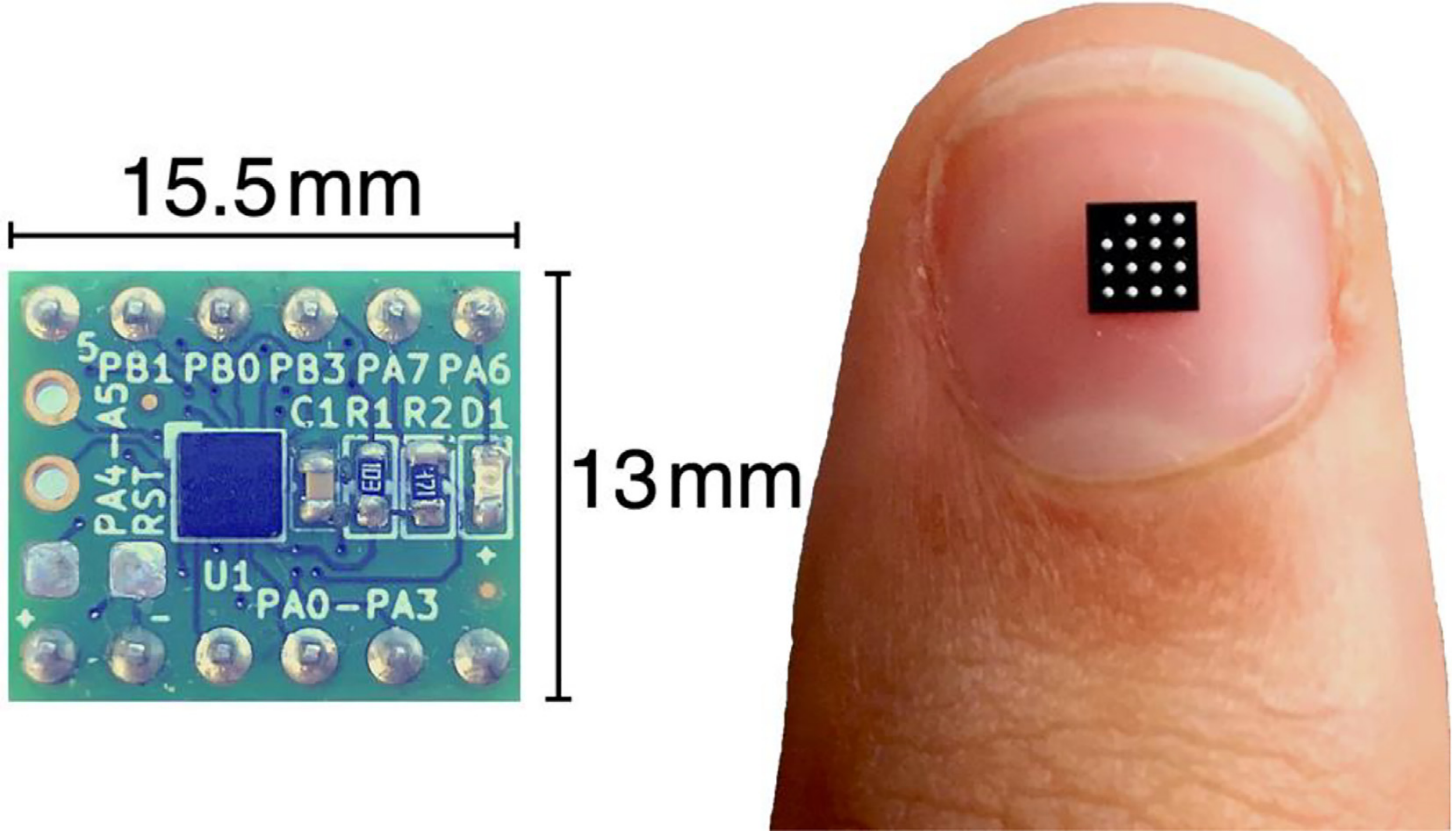
Conceived UFBGA *µ*-breakout PCB. The PCB area is only 15*.*5 mm *×* 13 mm.

In Fig. 3, the developed *µ*-breakout board is presented. The breakout board has practical value within electrical, instrumentation and biomedical engineering, and also with educational and learning activities for students, including IoT and cyber-physical systems.

## Design files

According to [13], the number of minimal routing layers needed to fully break out the BGA signal pins is estimated by:

(1) $$\text{Layers}=\frac{\text{Signals}}{\text{Routing}\;\text{Channels}\times \text{Routes per}\;\text{Channel}}$$

Since the ATtiny20-CCU has only 15 BGA balls and 12 general purpose I/Os, 80% of the number of BGA balls are associated with digital signals. The routing channels are defined as the routing paths out of the BGA area, i.e., the number of BGA balls of one side minus 1, multiplying by the 4 sides of the package. The routes per channel are the number of routed signals between BGA pads, usually one for the ATtiny20-CCU UFBGA-15. From (1), the minimum number of Layers is: 12/(12 *×* 1) = 1. Hence, 2-PCB-layers were chosen for the prototype design, to facilitate PCB routing and reduce the board size.

The design files for the developed board are provided in Table 1. The *µ*-breakout PCB was designed with KiCad EDA [14], [15], a cross platform and open-source electronics design automation suite. The SCH file allows to automatically generate a Netlist file, which is mandatory for the board design and to perform the PCB design rules check. Before the PCB design it is also necessary to assign the footprints to the schematic symbols. The Gerber files can be automatically generated from the PCB file, including the map and drill files. The fabrication files (Bill of Materials (BOM), Gerber and the Footprint Position) can then be sent to a PCB/PCBA manufacturer. The compiled and uploaded C code, to test the board (Code to test PCB.C), makes the LED D1 (pin PA6) initially blink every 0.1 s. The PA6 logic is inverted.

**Table 1:** Design files for the UFBGA *µ*-board.

Design filename		License	Location of the file
Board schematics	SCH file	CC-BY-SA 4.0	https://doi.org/10.17605/OSF.IO/YG653
Schematics for test	SCH file	CC-BY-SA 4.0	https://doi.org/10.17605/OSF.IO/YG653
Board PCB	PCB file	CC-BY-SA 4.0	https://doi.org/10.17605/OSF.IO/YG653
Code to test PCB	C file	CC-BY-SA 4.0	https://doi.org/10.17605/OSF.IO/YG653
Schematics for instrum.	SCH file	CC-BY-SA 4.0	https://doi.org/10.17605/OSF.IO/YG653
Instrumentation applic.	C file	CC-BY-SA 4.0	https://doi.org/10.17605/OSF.IO/YG653

**Fig. 4:**
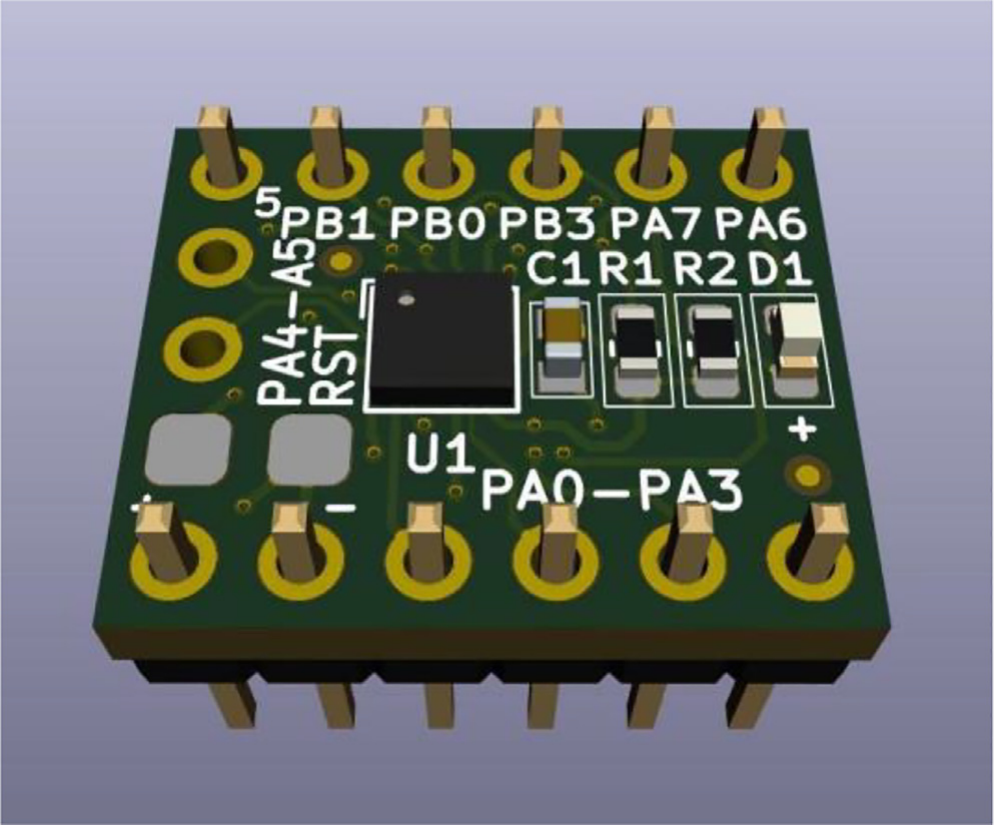
3D visualization of the UFBGA *µ*-board (front side).

**Fig. 5:**
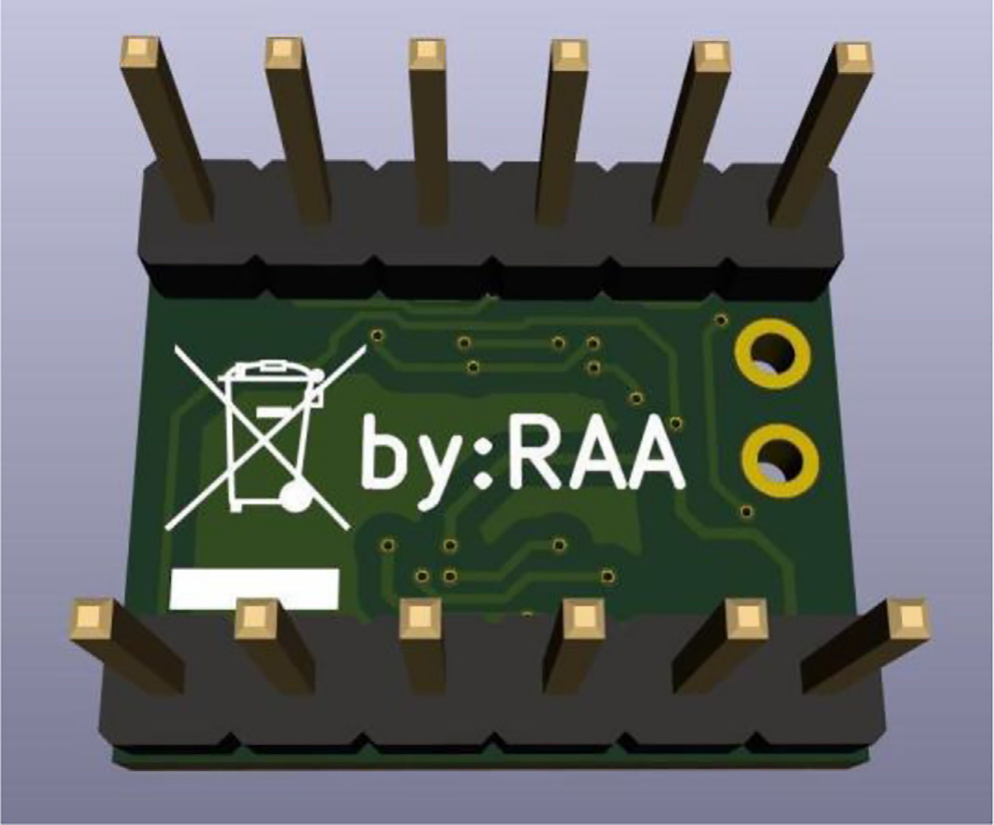
3D visualization of the UFBGA *µ*-board (bottom side).

The KiCad integrated 3D Viewer allows to instantaneous inspect the PCB design in a full interactive 3D view, allowing the designer to rotate and pan around, to easily inspect all the details. In Fig. 4, Fig. 5 are, respectively, presented the KiCad 3D views of the UFBGA *µ*-breakout board front and bottom sides.

## Validation and characterization

For the C test sketch to run, the ATtiny20-CCU was programmed without bootloader, using the Atmel-ICE programmer [16] and the Microchip Studio integrated development free platform [17]. The programming procedure is performed by accessing the three TPI board pins (PB1:TPIDATA, PB0:TPICLK and PB3:RESET). As an Atmel-ICE substitute, the Microchip Atmel AVRISP MKII in-system programmer, or the USBasp [18] low-cost programmer with the latest firmware can also be employed to upload the AVR compiled C code, using the command line free utility Avrdude [19].

## Bill of materials

The Bill of Materials (BOM) for the open-source UFBGA *µ*-board is provided in Table 2. The components cost, excluding the freight cost (20 €), is low, approximately 2*.*31 €. All components can be found at MOUSER Electronics, a global distributor of semiconductors and electronic components, or at Amazon. The components price can be considerably lower if large quantities of all components are purchased in a single batch order, minimizing the shipping cost.

**Table 2:** Bill of materials for the UFBGA *µ*-board.

Component	Qty. Un. cost	Tot. cost	Source of materials	Material type
U1 ATtiny20-CCU	11*.*1882 €	1*.*1882 €	Mouser: 556-ATTINY20-CCU	Electronics
C1 Capacitor 0.1uF	10*.*7601 €	0*.*7601 €	Mouser: 581-06033C104J4Z2A	Electronics
D1 Red LED	10*.*1562 €	0*.*1562 €	Mouser: 710-150060SS75000	Electronics
R1 Resistor 10 kΩ	10*.*1046 €	0*.*1046 €	Mouser: 603-RC0603JR-0710KL	Electronics
R2 Resistor 470 Ω	10*.*1046 €	0*.*1046 €	Mouser: 603-AC0603JR-13470RL	Electronics

In the Printed Circuit Board (PCB) fabrication, Electroless Nickel-Immersion Gold (ENIG) consists of a flat, solderable, metallic finish, that improves solderability and protects the copper from oxidation. In this industrial fabrication method used, the vias and the surfaces have an electroless Nickel layer applied to the copper. Next, it is given a thin Gold finish to prevent Nickel oxidation and to enhance solderability. The main benefits of the ENIG PCB technology are:

• Outstanding contact surfaces and higher solder joint reliability;
• Industry-grade manufactured circuit boards;
• Outstanding visual aspect;
• Robust protection for harsh environmental conditions.

Since the PCB size is only 15*.*5 *×* 13 mm, the PCB production cost of each board, from sending the Gerber files generated by KiCad to a typical PCB manufacturer, was down to 1*.*1139 €, for each board. Hence, the total cost of one UFBGA *µ*-board and the necessary electronics components was 3*.*4275 €. This cost does not include the components assembly and soldering costs (which are around 1*.*5 € for a single complete board). To reduce even more the UFBGA *µ*-board price, it is possible to design a multi-board, using the PCB Panelizer & Gerber freeware tool [20]. With this design methodology, the price of a single UFBGA *µ*-board can decrease to approximately 4 €, because a total of 90 UFBGA *µ*-boards can be produced in a single A4-sized PCB.

**Fig. 6:**
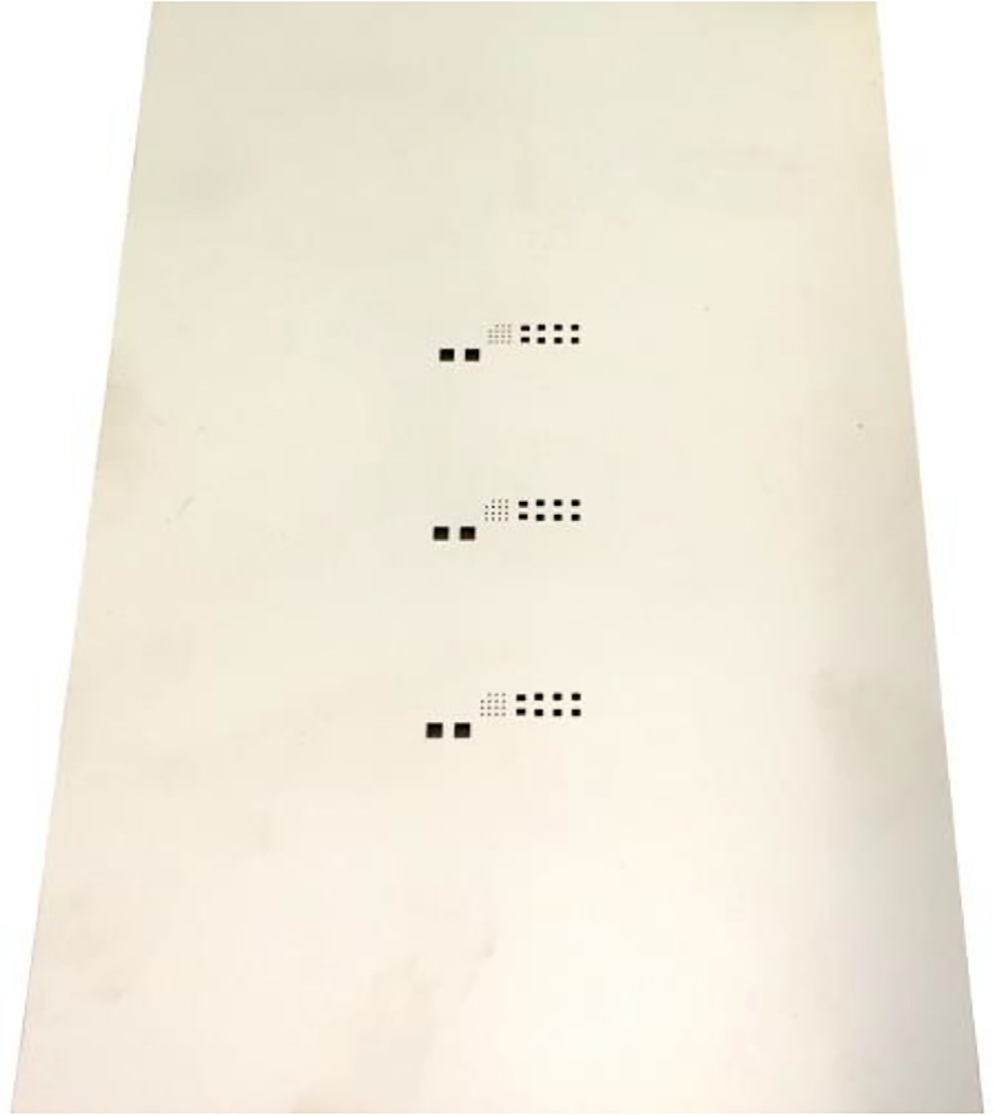
Stainless laser-cut steel stencil, to produce 3 UFBGA *µ*-breakout boards.

In Fig. 6, the stainless-steel stencil used for the solder paste dispensing and the components placement is presented. The price of the stencil production at the PCB manufacturer was 12*.*13 €. The Japanese-made 120 µm stainless-steel stencil with an electro-polished finish, allows the simultaneous soldering production of three UFBGA *µ*-boards.

## Build instructions

**Fig. 7:**
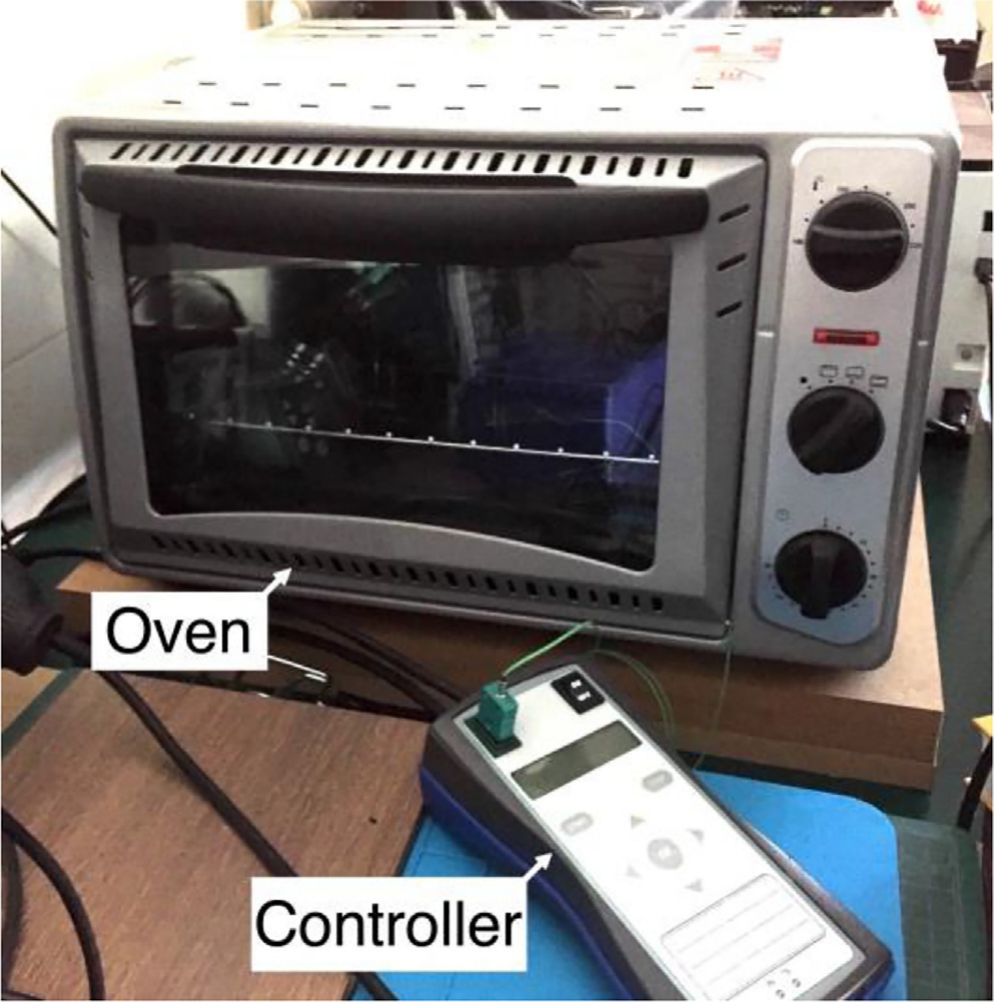
Reflow oven with appropriate controller.

There are two main approaches to solder the electronic Surface Mount Device (SMD)/BGA compo- nents in the PCB breakout board. The first approach, DIY (Do-It-Yourself), is to use high quality solder paste, a stencil and a reflow oven (Fig. 7).

After the solder paste is applied into the stencil holes, the components are placed manually, with a pair of tweezers, achieving precise soldering positioning. An SMD IC vacuum pick up suction pen can also be employed, as well as a SMT pick & place machine. A reflow oven is then used to heat the solder paste, and to perform the soldering process of the SMD and BGA electronic components to the circuit board. The reflow oven can be a standard or convection system with appropriate controller, or an infrared IC heater.

In this production process, the soldering temperature is the most important parameter. A temperature sensor is attached to the board to be soldered, using a wire. The reflow controller regulates the reflow oven, so that times and temperatures are maintained for the optimum soldering results. If the soldering temperature on the printed circuit board is achieved when the melting point can be observed, soldering is to be completed. The reflow controller, with a temperature sensor, controls the reflow oven according to the set-point temperature curve/profile. The soldering process parameters, such as the temperature and the soldering phase are monitored in the controller LCD (Liquid Crystal Display), and can be tuned directly from the controller panel, or via USB (Universal Serial Bus). It is also possible to build and store different temperature profiles, for several PCB components and solder paste.

**Fig. 8:**
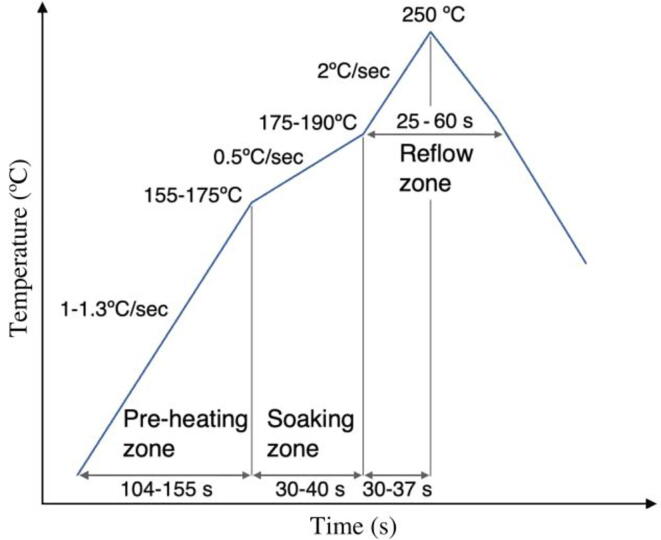
Typical UFBGA *µ*-board temperature profile, using NO-Clean, Lead-free solder pastes.

Before the soldering process, a BGA rework automatic machine (ex: the BGA Shuttle Star SV560) [21] can also be used to place and correctly align the ATtiny20-CCU chip in the PCB. These BGA rework machines are expensive, also requiring training to operate them. Since the ATtiny20-CCU ball pitch is relatively large (0*.*65 mm), this UFBGA-15 package can also be placed manually in the PCB, without the need for a BGA automatic machine, using for example a pair of tweezers. To obtain high-quality soldering results, some basic rules must be followed:

• The solder paste should be conserved at a cool temperature, typically near 10°C. A low cost, dedicated refrigerator/cooler (not for food) can be used, to store at 10°C the solder paste;
• The stencil must be exactly aligned to the pad patterns;
• The solder paste, after leaving the refrigerator, should reach ambient temperature before being applied, in a straight line, parallel to a chosen side of the stencil;
• Add and place the components with a pair of tweezers, or a suction pen/device;
• The reflow controller controls the reflow oven. For obtaining the best soldering results, temperatures and times must be previously settled;

In Fig. 8, a typical temperature profile for the UFBGA *µ*-board is depicted.

The PCB board, with the electronic components, are increasingly heated at the pre-heat stage. This procedure will prevent the occurrence of mechanical stress. After the pre-heat stage, the temperature will increase just below the soldering temperature (soaking phase) and the solder paste volatile elements vanish without causing blistering. The next stage is to heat the reflow oven to reach the soldering temperature (reflow phase), allowing the solder paste to liquefy and the components to adhere to the PCB pads. The soldering process finishes when the cool down ends. A second place & soldering methodology, not the DIY (Do-It-Yourself) way, consists in sending the Gerber files, the Bill of Materials (BOM) file and the pick & place file to a PCBA (Printed Circuit Board Assembly) manufacturer. All these files are automatically generated by KiCad, the PCB design freeware tool used. Many industrial PCBA manufacturers allow to register and send online the PCB files for small batch PCB manufacturing. Several in-website customized PCB production options are allowed, such as the number of layers, the PCB material, thickness, and the surface finish (ex: ENIG). In the PCB Assembly (PCBA) turnkey option, the PCBA manufacturer supplies all or some of the electronic parts. A contract/quote may be previously negotiated, for high volume PCB assembly services.

Concerning the Ball Grid Array (BGA) inspection, it is not possible to visually inspect the PCB soldering quality, since the solder balls are below the chip. Hence, the main BGA disadvantage is that the inspection, and also the rework, are relatively difficult to perform. Possible methods for BGA inspection are the electrical tests and also the boundary scan. A traditional electrical test allows to seek for open and short circuit defects. Boundary scan technology, depending on the inspection ports designed, provides access to each solder joint on the boundary connectors. However, both techniques only test the PCBA electrical reliability, and aren’t able to inspect the PCBA soldering quality. AXI (Automated X-ray Inspection) can solve the inspection problem in real-time large-scale production.

## Operation instructions

The developed UFBGA *µ*-breakout board can be programmed without the need of an internal ATtiny20-CCU bootloader, using the low cost USBasp programmer (available at eBay for only 1*.*76 €), or the Atmel-ICE programmer for Atmel AVR microcontrollers. Because the ATtiny20 has only 2 K bytes of programmable flash memory, an eventual bootloader for this low-cost microcontroller may reduce in more than 30 %, the available programming memory, which is not recommended.

**Fig. 9:**
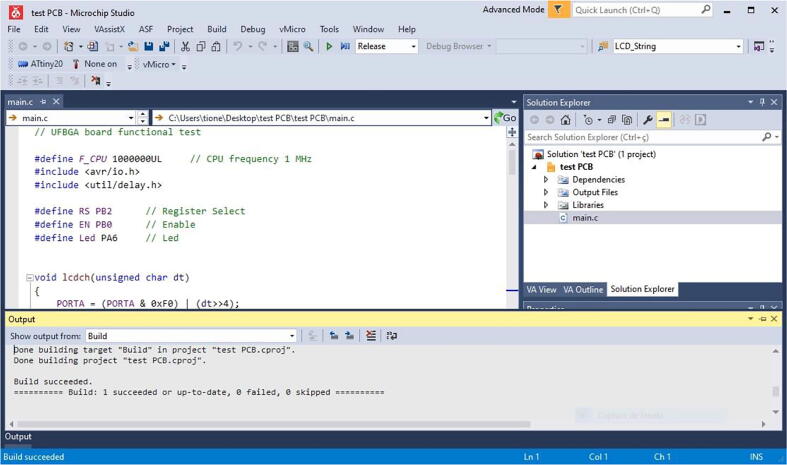
The Microchip Studio (version 7.0.2542) integrated development freeware platform [22].

**Fig. 10:**
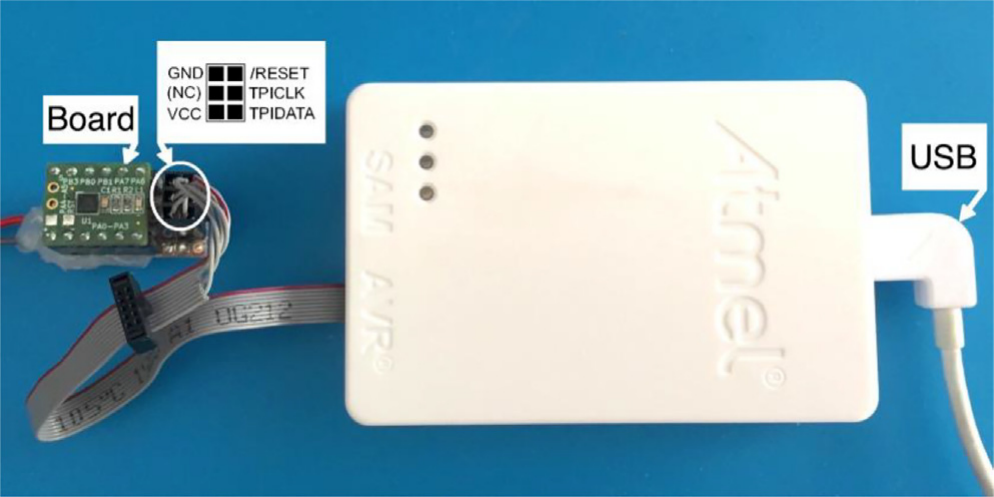
Atmel-ICE TPI pin connections.

**Fig. 11:**
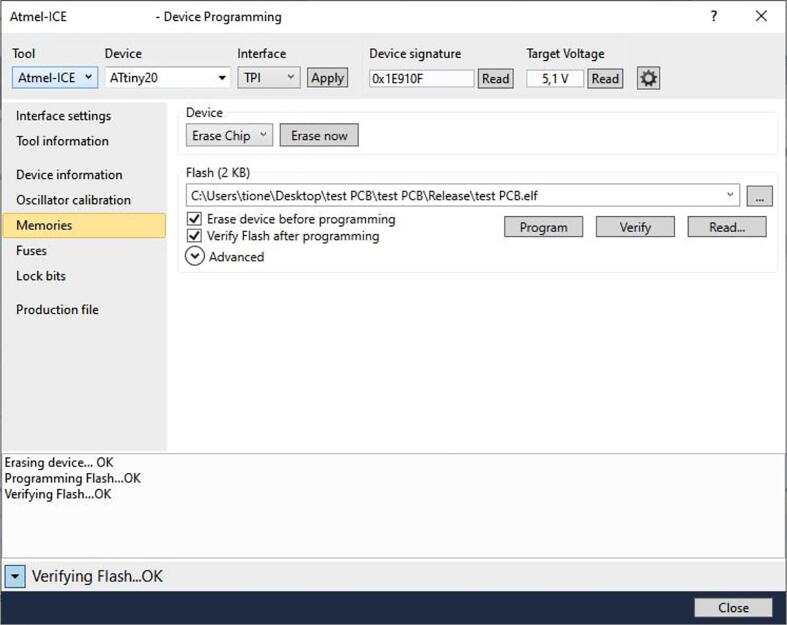
Programming the ATtiny20-CCU *µ*-breakout board, with Microchip Studio.

The UFBGA *µ*-breakout board was previously programmed with the compiled test code, through the Atmel Tiny Programming Interface (TPI), using the Microchip Studio integrated development freeware platform [17] and the Atmel-ICE programmer [16], as shown in Fig. 9, Fig. 10, Fig. 11. The TPI header pins, depicted in Fig. 10, are: TPIDATA:PB1; TPICLK:PB0; RST:PB3; VCC=+5V and GND. After programming, the UFBGA *µ*-breakout board is then inserted into the breadboard and the pin PB1 will operate as an input, connecting to a push-button.

**Fig. 12:**
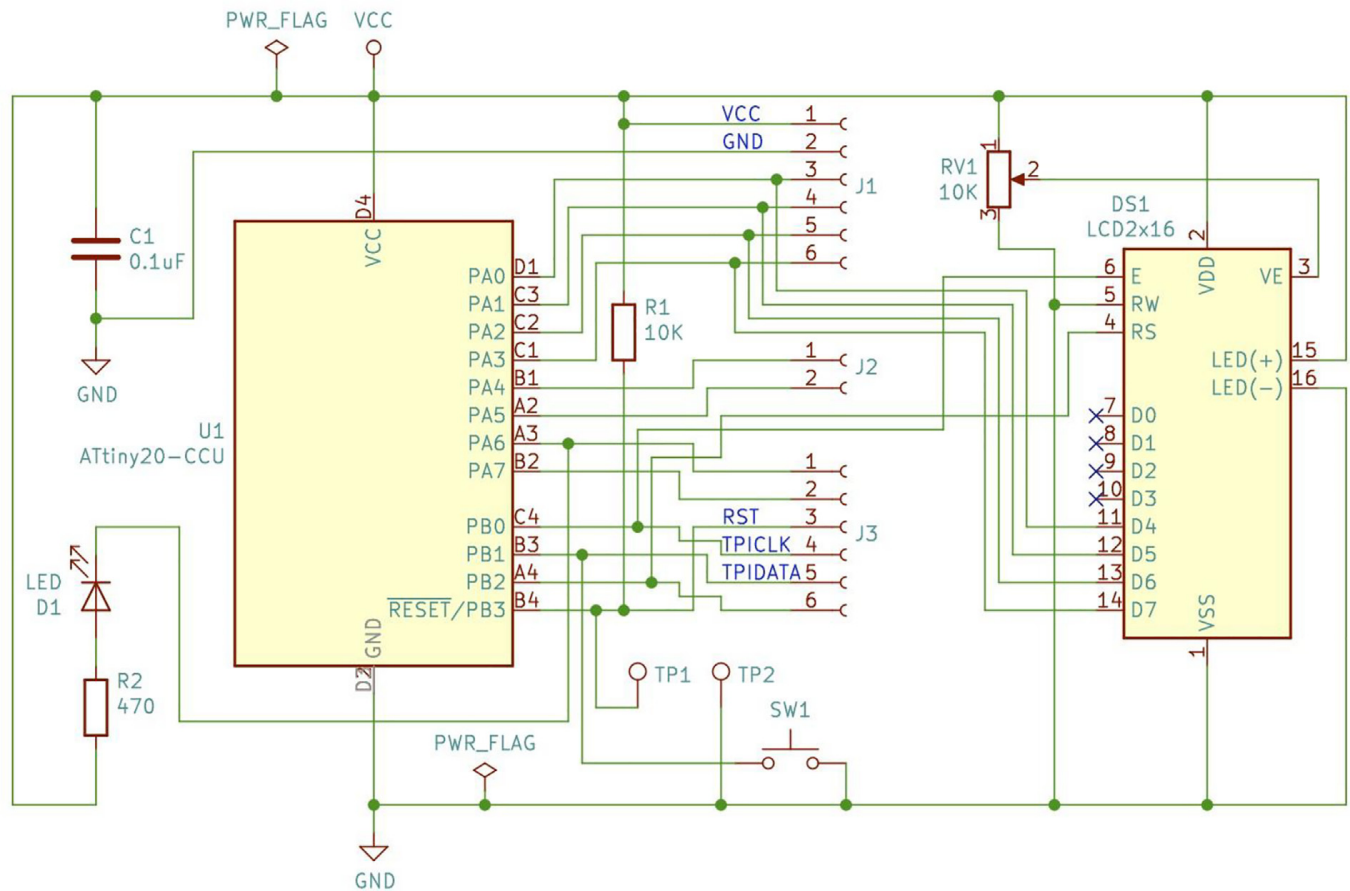
UFBGA *µ*-breakout board circuit test, with the LCD 2 *×* 16, the LED D1 and the push-button SW1.

**Fig. 13:**
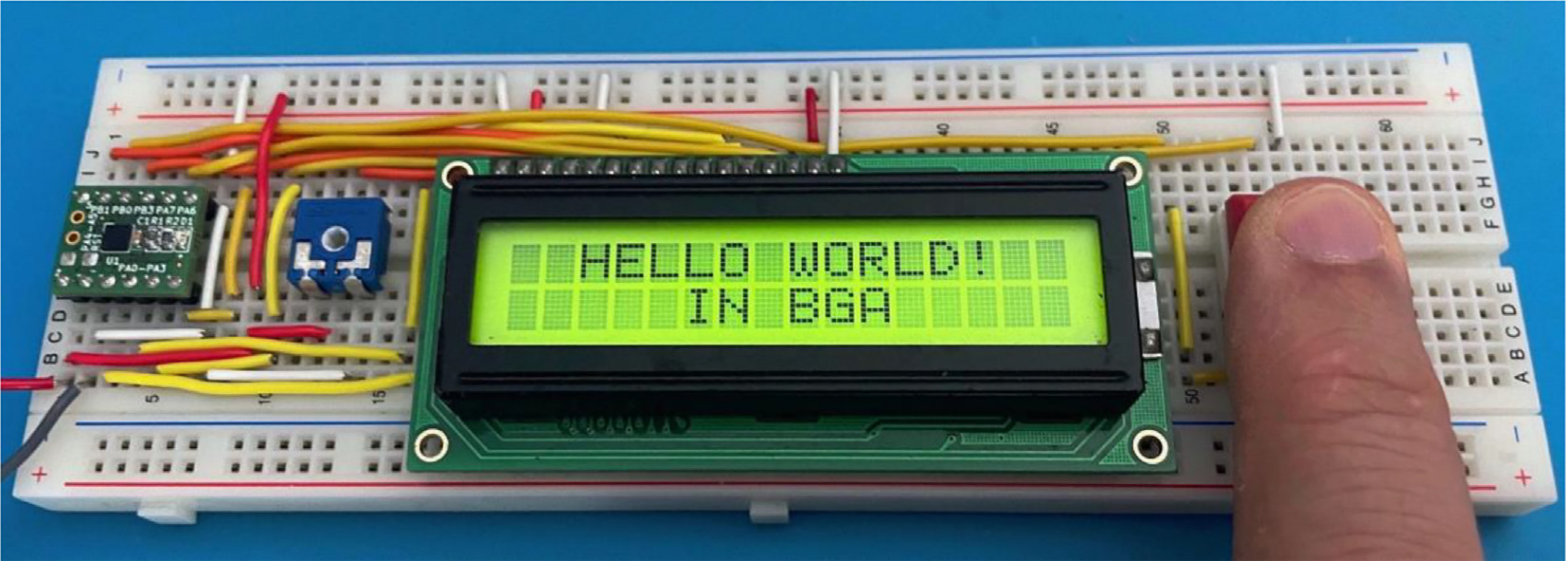
UFBGA *µ*-breakout board functional test.

In Fig. 12, the self-test circuit for the UFBGA *µ*-breakout board is presented. The developed *µ*-breakout board is attached into the breadboard, connecting to a standard LCD 2 *×* 16 and a push-button (SW1). The LCD used has eight data pins (D0 to D7) and three control pins (E, RW and RS). The communication between the ATtiny20-CCU and the LCD 2 *×* 16 is performed using the 4-bit mode. Hence, the PA0 to PA3 microcontroller outputs are connected to the D4 to D7 LCD inputs. The initial C language test code (Code to test PCB, in Table 1) is a blinking action at the internal LED D1 (PA6 output) in serial with the R2 resistor, while the push-button SW1 (PB1 input) is not being pressed. A LP-2800 logic probe [23] is used to check the logic states at the PA6 output, and at the PB1 push-button input. When the SW1 push-button is maintained pressed by the human operator, the LCD 2 *×* 16 displays the following message: “*HELLO WORLD! IN BGA*” and the PA6 output is set to the logic state “HIGH”, as shown in Fig. 13. When PA6 is “HIGH”, the LED D1 is off.

The ATtiny20-CCU microcontroller has an advanced RISC (Reduced Instruction Set Computer) architecture with 112 instructions, most single clock cycle executed, allowing at 12 MHz a throughput up to 12 MIPS (Million Instructions per Second) [12]. The functional test code (Code to test PCB C file, Table 1) uses 17,4% of the available program memory (2 K bytes). The maximum device throughput at 1 MHz is 1 MIPS. At this clock frequency (1 MHz) the mean electric current consumption of the UFBGA *µ*-board with the internal LED D1, when performing the functional test, is 4*.*3 mA (the LCD electric current is not considered).

**Fig. 14:**
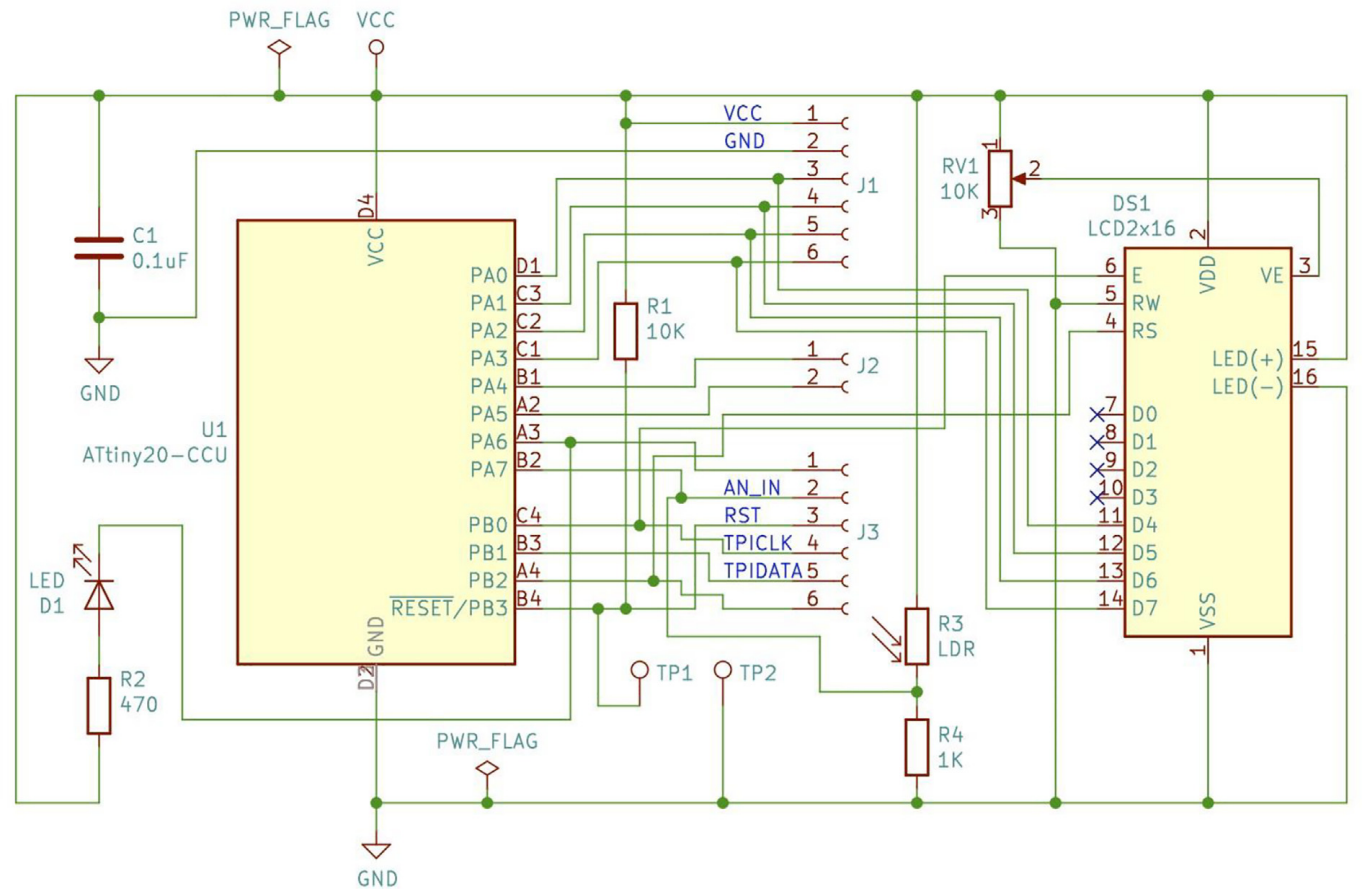
UFBGA *µ*-breakout board circuit for instrumentation application.

**Fig. 15:**
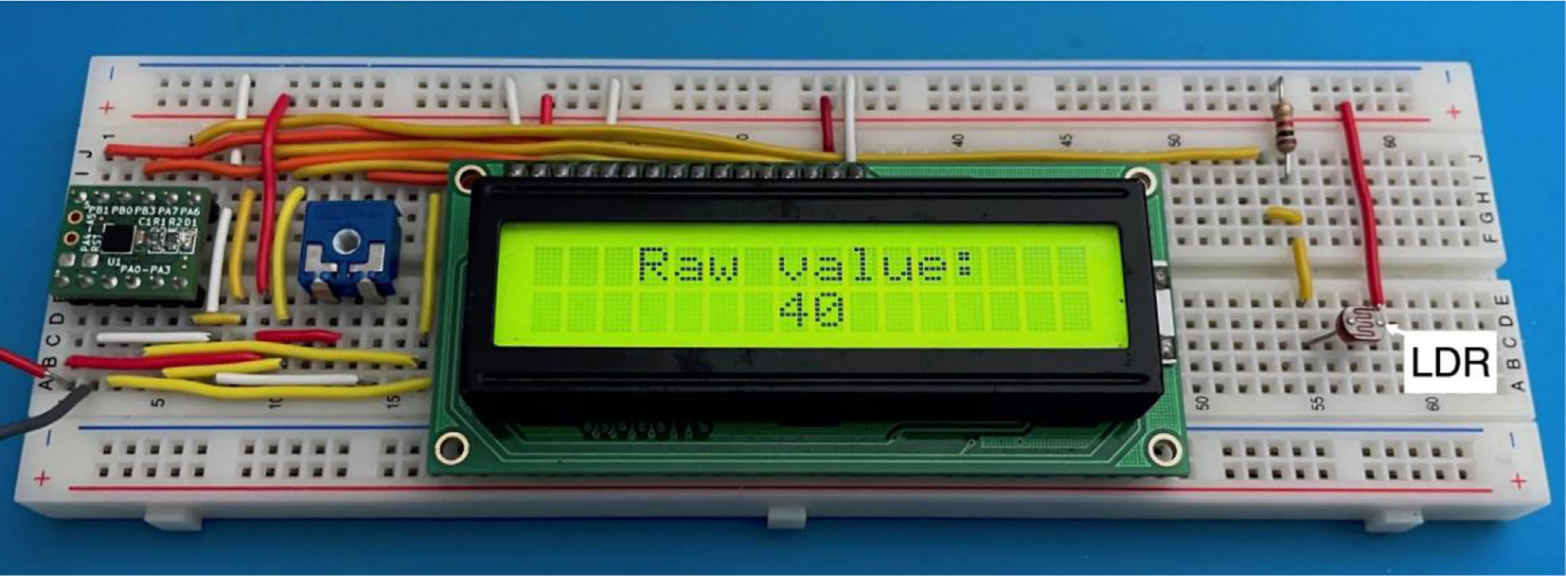
UFBGA *µ*-breakout for the LDR instrumentation application.

In this work, an instrumentation application example was also made, using a similar GL5528 Light Dependent Resistor (LDR), to measure the ambient light intensity. In Fig. 14, the correspondent instrumentation circuit is depicted. The LDR photoresistor circuit output connects to the analogue input AN_IN (pin PA7). The light intensity raw values are shown, in real-time, in the LCD (Fig. 15), varying between 0 and 255. The ATtiny20-CCU ADC was configured with 8-bit resolution. The compiled code for the instrumentation application (Table 1) uses 35.3% of the available program memory. The electric current consumption of the UFBGA *µ*-board in the instrumentation application with the LDR varies between 2*.*3 mA (dark) and 6*.*5 mA (shiny).

## Future developments

For future developments, a PCB interface board that contains the Atmel-ICE 6-pin connector, for ISP/debugWIRE/PDI/aWire/TPI interface, can also be made. The interface board is to be directly attached to the *µ*-UFBGA board, for programming the ATtiny20-CCU AVR microcontroller via TPI. A reverse polarity over-current protection can be also implemented, using a SMD Schottky diode, a SMD fuse, or a SMD PTC (Positive Temperature Coefficient) resettable fuse, placed in serial with + VCC.

A PCB castellated holes version of the *µ*-UFBGA board may also be designed and produced. The current 2*.*54 mm pin DIP version is to be mainly used for breadboard experiments, educational Instrumentation and/or Biomedical projects. Many other hardware projects, where the demand for low size and power consumption are required, can also be accomplished using the UFBGA packaging technology. The castellated holes PCB version is intended to be SMT soldered to a shield PCB, extending the board capabilities. The UFBGA *µ*-board castellated holes final size will be only 13*.*0 *×* 10*.*1 mm. The PCB thickness will also be reduced from 1*.*6 mm to 1 mm.

The UFBGA *µ*-breakout board can be connected to several sensors/actuators for wearable instrumentation, biomedical and IoT applications, or to cyber-physical systems, using the available digital inputs/outputs, analogue inputs, or SPI interface.

Efficient optimized code programming is required, since the ATtiny20-CCU microcontroller program memory is confined to 2 K bytes. Given this fact, it does not allow the implementation of some complex C functions which occupy relevant amount of program memory, such as the *sprintf* () function. Because the ATtiny20-CCU does not have internal UART (Universal Asynchronous Receiver-Transmitter), an optimized ATtiny20-CCU virtual UART library can be developed from scratch, in a future work. The ATtiny20-CCU virtual UART code could be further optimized to fill within 2 K bytes if it focuses mainly on the transmission of sensor raw data. The raw information should be processed by a software application running on a portable mobile device with Bluetooth and a human-friendly graphical interface.

For more powerful computational tasks embedded in wearable devices, the Atmel 32-bit Cortex- M0 SAM D1x/2x MCUs family is an available option, also in UFBGA technology.

## Human and animal rights

The authors declare that no human or animal subjects were used in this study.

## Declaration of Competing Interest

The authors declare that they have no known competing financial interests or personal relationships that could have appeared to influence the work reported in this paper.

## Data Availability

Data available in data repository at https://doi.org/10.17605/OSF.IO/YG653.
